# Consumer Feedback following Participation in a Family-Based Intervention for Youth Mental Health

**DOI:** 10.1155/2012/235646

**Published:** 2012-09-05

**Authors:** Andrew J. Lewis, Melanie D. Bertino, Narelle Robertson, Tess Knight, John W. Toumbourou

**Affiliations:** Centre for Mental Health and Wellbeing Research, School of Psychology, Faculty of Health, Deakin University, Melbourne, VIC 3125, Australia

## Abstract

*Background*. This paper presents findings derived from consumer feedback, following a multicentre randomised controlled trial for adolescent mental health problems and substance misuse. The paper focuses on the implementation of a family-based intervention, including fidelity of delivery, family members' experiences, and their suggestions for program improvements. *Methods*. Qualitative and quantitative data (*n* = 21) were drawn from the Deakin Family Options trial consumer focus groups, which occurred six months after the completion of the trial. Consumer focus groups were held in both metropolitan and regional locations in Victoria, Australia. *Findings*. Overall reductions in parental isolation, increases in parental self-care, and increased separation/individuation were the key therapeutic features of the intervention. Sharing family experiences with other parents was a key supportive factor, which improved parenting confidence and efficacy and potentially reduced family conflict. Consumer feedback also led to further development of the intervention, with a greater focus on aiding parents to engage adolescents in services and addressing family factors related to adolescent's mood and anxiety symptoms. *Conclusions*. Participant feedback provides valuable qualitative data, to monitor the fidelity of treatment implementation within a trial, to confirm predictions about the effective mechanisms of an intervention, and to inform the development of new interventions.

## 1. Introduction

There is an increasing recognition of the need for early identification and intervention for youth mental health problems such as depression, anxiety, and substance use. These problems are of growing community concern given their high prevalence [1–3]. The field of youth mental health research now faces a major task of translating a growing body of research into effective clinical practice and service development [4, 5]. Youth mental health disorders are associated with increased health problems, and with problems in family functioning [6–10]. Recent estimates of treatment for depression suggest that only 20% to 30% of the years lived with disability due to depression are averted by current treatment programs, suggesting room for substantial improvement in either service delivery or effective prevention of new cases of depression [11, 12]. One means of enhancing the efficacy of interventions is to shift the focus from outcomes to issues of implementation which arise in the translation of clinical findings to service delivery systems. In doing so interventions can be developed in directions which are well aligned with relevant government policy, as well as being acceptable and engaging for client groups.

The current paper reports on implementation issues in the “Deakin Family Options” (DFO) multicentre randomised controlled trial (RCT) which compared two interventions for youth depression, anxiety, and substance use. We report qualitative information gathered from participants following the completion of their psychological treatment in the DFO trial. The two treatments in the trial were both designed to reduce adolescent depression and substance use and included an individual CBT program and a family-based program known as “BEST-Plus” (Behaviour Exchange Systems Training for parents “Plus” youth) [13]. The aim of the paper is to evaluate participant feedback on the intervention experience, particularly within BEST-Plus; in order to assess treatment fidelity, effective treatment mechanisms, and potential future program modifications.

Traditionally, RCTs have been concerned with clinical efficacy. However, evaluation of the real world effectiveness of interventions delivered in community settings requires examination of direct feedback from those undertaking the interventions [14, 15]. In addition to the traditional RCT outcomes such as quantitative statistical analyses of group differences, collecting, and analysing qualitative data on individual consumer experiences can provide useful insights. Qualitative data collected from participants undergoing psychological treatments can be used to aid in the translation of research into practice. By capturing the lived experiences of study participants, researchers can gather information on the perceived mechanisms and barriers to change, and suggest further ideas for improving and enhancing interventions [16, 17]. 

The DFO trial was delivered in a community setting and designed in a manner that attempted to enhance its relevance to parents in the general community who had concerns about the mental health of their adolescents. As such, the DFO trial adopted inclusion criteria which were directly aligned with clinical referral patterns by recruiting young people (aged 12 to 25 years) if they presented with depression and/or anxiety and/or substance use problems. Both the age range of participants and the presenting issues were as inclusive as possible to fit directly with the Australian service delivery system for youth mental health. The purpose of the trial was to evaluate the relative efficacy of three treatments: (1) a family-based treatment program (BEST-Plus); (2) a cognitive-behavioural therapy (CBT), individual treatment program for the youth; (3) receiving both interventions. This paper primarily focuses on families' experiences of BEST-Plus. Much of the data from the consumer focus groups is based on these parent's experiences in the BEST-Plus groups, including their feedback and insights into the perceived mechanisms and barriers for change in themselves and their young person. 

The BEST-Plus program is based on an earlier version of the program known simply as BEST, developed by Toumbourou, Bamberg, and colleagues [18]. It was initially developed as a professionally led, multifamily group education program for parents, with content focussed on alcohol and drug use by adolescents. The BEST program was shown to reduce parental mental health symptoms and family stresses [19]. To increase program efficacy, the second stage of development (BEST-Plus) included all family members and focused on inviting siblings, who joined their parents in the group for the final four weeks of the eight-week program. Evaluations showed that additional positive changes in the family system were produced in mental health and stress symptoms, family cohesion, and increases in action by young people to address their substance use and thus improve their mental health [20, 21]. 

Family-based interventions are less common in the mental health system than psychological therapies focussed on individuals. However, family-based interventions have a number of potential advantages for adolescents in terms of engagement and capacity to address the impact of mental health problems in the family's transition across key developmental periods. There are many circumstances where, for a variety of reasons, an adolescent refuses to participate in a mental health service; this poor uptake of services by youth is extremely common in current Australian youth mental health services [22]. One of the primary aims of the current study was to evaluate how a family-based intervention model can be used to address legitimate concerns raised by parents about the mental health of their adolescent to the benefit of the family as a whole.

Our research questions for this evaluation focused on participant's responses to the BEST-Plus interventions. We were interested in examining how these groups might have benefited parents, and the mechanisms which participants identified as being effective. We were also interested in attempting to understand how family-based groups helped parents to address the mental health needs of their young person, and whether these mechanisms and interventions had been faithful to the treatment manual for BEST-Plus. Finally, we were interested in new ideas that parents in particular had to improve the efficacy of this intervention approach.

## 2. Method 

### 2.1. Study Design and Sample

While the overarching DFO study was designed as a RCT, the current paper reports mainly on the qualitative data collected from participants during focus groups held 6 months after their treatment in the DFO study. Participants were invited to the focus groups if they completed one of the trial interventions. The experimental treatment was the BEST-Plus program which is a fully manualised treatment. It consists of an eight-week, professionally-led group program designed to assist parents concerned with youth substance use-related problems. The parent/s receive 4 sessions of weekly intervention and then the parent/s, sibling/s, and young person complete 4 sessions of a weekly intervention together, where the family members are willing to attend. The control condition was the CBT intervention for the young person alone. Only one participant in the focus groups received the combined treatment arm (such that they received both the BEST-Plus intervention for the family, and the individual CBT treatment for the young person), and therefore, these results were combined with the results of the rest of the attendees who had participated in the BEST-Plus treatment arm. All interventions were delivered by trained and supervised clinical psychology trainees who were undertaking Masters level training. All therapists received supervision, training; and therapy manuals. A total, of *n* = 186 individuals participated in the DFO trial which consisted of *n* = 71 adolescents (38.2%); *n* = 70 mothers (37.6%); *n* = 29 fathers (15.6%); *n* = 13 siblings (7.0%) and *n* = 3 step-parents. In total *n* = 86 family units were recruited, of which 13 families participated in the focus groups. Participants in focus groups were compensated with vouchers for their time. Compared to the families who did not participate in the focus groups, the current sample were not significantly different in level of family income, level of education, or in terms of the type of family member who participated (mother or father), but did differ significantly in terms of being more likely to be intact families (married) and more likely to have completed all study questionnaires.

### 2.2. Measures

A set of ten questions were used in the focus groups as prompts for group discussion. These questions were as follows: (Q1) What were the most valuable aspects of being a participant in the BEST-Plus group? (Q2) Were there any negative aspects of being a participant in the BEST-Plus group? (Q3) How did your initial expectations relate to what the BEST-Plus group delivered for you? (Q4) Has what you learned from the group impacted the way you parent your young person? (If so, how?) (Q5) Is there anything you would like to see included or changed to improve the program? (Q6) Would you recommend the program to other parents? (Q7) When invited, did your young person or other children in the family attend the BEST-Plus group at session four; if so, what might have helped to allow the young person to attend? (Q8) What aspects of the group did you implement in your family life? (Q9) What additional services, if any, have you accessed since your involvement in the study? (Q10) How are things at present in your family?

Quantitative measures were also administered. At the commencement of focus group meetings, participants were also asked to fill out a brief feedback survey. This consisted of questions concerning the intervention received and the level of satisfaction with (1) the intervention received, (2) improvements in your family/home life since completing the program (3) overall satisfaction with the experience of the Deakin Family Options program; each rated on a scale of 1 to 10, where 10 represents complete satisfaction. Participants were also asked whether they were still implementing the skills and knowledge gained from the program in their daily lives and “Did you feel the program adequately addressed your needs?” and “Would you recommend participating in this program to a friend experiencing similar problems” with Yes/No response options.

### 2.3. Procedures

Three consumer-reference groups were run at the end of 2011 with 7 or 8 participants in each group. Groups were facilitated by the same people that had facilitated the BEST-Plus group. The focus groups ran for 1.5 hours. The focus groups were recorded with consent and transcribed and verified by two observing researchers. Participants were also asked to fill out a brief feedback survey. Most participants (*n* = 21) in the focus groups were referring to the time they spent in the BEST-Plus groups. In one focus group, two young people from the same family attended with their parents. 

### 2.4. Data Analysis

Descriptive statistics were used to report quantitative data derived from a consumer satisfaction survey and data collected on treatment engagement. The analytical approach we took to the qualitative data was broadly phenomenological in that the emphasis was on the subjective experience and personal interpretation. In line with the phenomenological theory of qualitative research, we were particularly interested in allowing the voices to be heard in order to gain insight into what motivated and engaged the participants [23, 24]. The interviews were transcribed verbatim for analysis, which initially entailed reading the transcripts several times to capture the essence of the data. This process was completed by two members of the research team who then reread the text to draw out emerging themes or meanings embedded in the participants words and discussed their findings to reach consensus on any of the points where disagreement occurred.

## 3. Findings

Sample characteristics of participants in the reference groups are presented in Table 1.

### 3.1. Engagement of Young People in Mental Health Services

All participants in the focus groups received the BEST-Plus intervention and engagement rates are presented in Table 2. Overall, 53% of participants engaged in a treatment offered to them after an assessment. Engagement in the present context refers to completing the majority of treatment sessions. This figure may seem low but includes many circumstances where a parent would agree to participate, that is, their adolescent randomised to enter the CBT service, but the young person in their family would refuse to participate. 

 As presented in Table 2, young people were disproportionately less likely to engage in treatment (i.e., 60–70%) versus parents (20–30%) who do not engage. This difference was statistically significant (*χ* ^2^(5) = 28.8, *P* < .001). It is also interesting to note that although a larger number of mothers than fathers presented for service within the study, those fathers who did present were more likely to be engaged in a given treatment.

### 3.2. Focus Group Themes

*We are not alone…*

Participants enjoyed the collegial atmosphere of the BEST-Plus groups where they felt that the group process and sharing of experiences helped them to feel that that they were “not alone”. Parents considered this to be helpful in that it showed them that other young people went through similar experiences. Participants also commented that they appreciated the safe space that was created by the facilitators so that they could talk and contribute their experiences. The contribution of their own understanding to try to help others in the group was also a key aspect of the group experience. Participants noted that groups worked well when they were very participatory, making the groups a “give-and-take” experience. Parents felt that they learned most from each other. The most common benefits that were mentioned in both focus groups were the advantage of being with others with similar issues, the support, and advice they were able to give and receive as well as not feeling so isolated and alone. This exchange of experiences and help, some participants felt, also helped in reducing levels of self-blame and guilt.

*I was just feeling so beaten up and battered…so coming here on my own and listening to the other stories of parents, their stories, I felt I wasn't as really as bad as I'd escalated it into my head…I just felt really secure and um, able to say what I felt and felt supported… and It is quite hard for me to let go.*

*It is their Journey*

A dominant theme that ran through both of the focus groups was learning to let go and allow the young person to take responsibility for their own life journey. The group had helped parents to understand the importance of separation and individuation in the family developmental process. The way this was expressed was in terms of the ability to “stand back and let go”. Parents described how prior to this realisation there was a sense of helplessness, not knowing what to do for, or how to be with their young person, and being constantly caught up in conflict with their young person. They found that by stepping back and allowing young people to experience consequences it helped to defuse the “weapons” (as one participant called them) that their child would use to provoke them. Parents considered that one of the key processes through which they changed their relationship with their adolescent was learning to act rather than always reacting to a situation. This suggested an increased confidence and a greater focus on more authoritative and proactive parenting. Parents also realised that such changes occurred as they experienced reduced levels of distress and anxiety. Letting go also gave rise to opportunities for parents to take time out and to consider their own needs.

*I felt that what we got out of the group was the letting go part and realising that it is their (the young person's) journey. I think we also approach things in a much different way than what we did… and Just more time out. More time out.*

*Self-Care*

The group spoke about the importance of self-care. It was suggested in the focus groups that self-care was something that parents were still implementing after the course had finished; that despite their situation, they recognised the need to look after themselves in order to better care for their child. This recognition emerged from the understanding that, no matter how much they wanted to help make everything better for their child, that ultimately their child needed to own his/her life and embark on that journey, the process was not always easy and the need to intervene often overwhelming. One woman related how her daughter would say “everythings ok … and then she'll tell me she's not great—and I'm just … my stomach drops”. In the past, what might have led the parent to want to step in and take over became a recognition that she needed to support her child, to be there, but in order to do that she needed to attend to her own well-being.

*But I had the chance to go away on my own, which was nice and think just about myself and I keep thinking about what (facilitator) said—that it's her journey and that's probably the most helpful thing I took out of it [the program]. It's her journey and I need to be there, but ultimately it's her life—it's not my life.*

*Metaphors to live by*

One of the notable features of the BEST-Plus program is the use of several metaphoric parables which are presented by group facilitators, often with an illustration, designed to evoke themes relevant to the key developmental processes and challenges facing a family during their children's adolescence.

*It sounds flippant, but it's funny to think that such a small diagram can put you in a mindset to think yes, we did launch off on our own when we were young, and kids have to do that…*

This quotation from a parent exemplifies the power of the metaphor. Having one's situation that seemed so insular likened to a familiar and shared situation helped participants picture their own world from a different perspective. Participants in the focus groups found the metaphors employed in the BEST-Plus groups gave them a new outlook that they were able to continue to employ. They still remembered them and still found them useful. The use of the metaphor tied their situation to something more positive, helped give them context, and make the situation more concrete.

*The Young person's perspective*

There were two young people from one family who attended the Melbourne focus group with their parents. One of the young people who had been through the CBT arm of the program reflected that the self-initiated effect of gaining independence by moving out of home had been the most significant thing for him. As well, both felt that they now addressed issues with their parents in a more upfront manner, which they felt was positive. They also felt that their parents had changed how they “dealt” with them.

*If I have a problem I address it now, like if they have a problem with me they address it straight up, we get it over with, so yeah it might be a bit confrontational but it gets it over and done with. *

The BEST-Plus program consists of eight sessions. In the first four sessions the focus is exclusively on the parents. In the final four sessions parents invite their children to attend. One of the issues raised by group attendees was the low-levels of participation of the young people in the second half of the BEST-Plus groups, although this was mitigated to an extent by some parents engaging their young people within family discussions about their attendance at the group. Such discussion was done primarily so that the young person would know that they were being proactive about finding solutions to the family challenges. This reflects a proactive change in parenting styles that was commented on by many participants. 

Many participants would also have liked some continuation of the group because they found the parental support to be very valuable. Some parents suggested that running parent support groups that they could transition into would be beneficial. Generally, participants mentioned that they had started the group with the idea of changing the behaviour of their young person. For many this initial goal had given way to parents thinking that the greatest benefit of the groups was rearranging how they parented and changing their ways of handling family situations and challenges.

*Program development*

Participants offered a number of suggestions on what needs to change to make the BEST-Plus program more relevant to their own and their adolescent's mental health. Parents commented that there was too much information in the groups focused on managing “externalising” behavioural issues such as violence and crime in their young people, and some parents with young people that had depressive or anxious children sometimes found that the information about challenging behaviour was less relevant to their situation.

*There was such a diverse range of problems in the group. I found that a lot of the problems and strategies were for behavioural issues where as we are dealing [with] mental illness. The group did not really cater for mental illness.*

However, parents generally acknowledged that they derived considerable benefit from the program. All of the participants of the focus group would recommend the Deakin Family Options program to others. Some even felt the BEST-Plus group was better than they had expected.

*One other thing I think I had, we approached another school counsellor and then another school counsellor and then we were referred onto 1, 2, 3 places so that's five lots of people, so I had very low expectations of actually like, anything actually engaging with our reality so I was a bit blown away that it did and it did it in a way, not quite the way we expected…*

*Participant direct recommendations*

(1) Weekly sessions should be longer; some participants felt that 2 hours was not long enough. Participants would have liked an extra half an hour or so to extend their discussions.
(2) Participants would like on-going support; the majority of the participants would have liked the group to continue beyond the 8-week program. Some considered follow-up sessions once a month would be valuable.
(3) There needs to be an improved balance in the focus on behavioural *versus* mental health issues. All participants felt that they had gained something from the BEST-Plus program, but parents whose young person had depressive and anxious disorders would have liked some of the weekly group focus directly on how to address these issues rather than spending too much time on behavioural and drug use issues.
(4) Earlier intervention was highly recommended. Interventions need to be offered to parents before major problems arise. Participants felt that having something set up as an early intervention would be very helpful to them. This would help them to address parenting and potential challenges with adolescents before the problems arise. Parents suggested that similar content would be helpful if it commenced in early primary school years and was offered within a school setting.

## 4. Results from the Feedback Survey

Results from the consumer satisfaction surveys completed on the same evening as the focus groups are presented in Table 3. The young person in attendance did not complete the questionnaire. Overall most participants were satisfied both with the intervention and their experience of the Deakin Family Options program. Satisfaction with the improvements in their family life since completing the program scored slightly lower. However, when asked the question, “would you recommend participating in this program to a friend experiencing similar problems?” all participants (*n* = 20) answered yes. The majority of parents felt that the Deakin Family Options program had adequately addressed their needs, for others, the lack of behavioural change by their young person impacted on their satisfaction with the program. No participant responded that the program had not addressed their needs at all. Nearly all (*n* = 20) participants were still implementing the skills and knowledge that they gained from the program in their daily life approximately six months after completing the intervention.

## 5. Discussion

There were four major themes that consistently came out of the focus groups. Participants pointed to the advantage of meeting with like-situated parents and being able to safely share their experiences under facilitation. The advantage of this was to break down the sense of isolation. From the weekly sessions, the participants felt they learned or relearned the skill of taking a step back from the situation. Acknowledging the responsibility their young person had to take for their own lives and actions was also a powerful therapeutic moment for many parents. This helped alleviate the guilt and sense of helplessness that was a common experience described by participants. Further, participants took away the idea of the importance of self-care. The role of metaphor within the program was also confirmed as a valuable element, helping parents to situate themselves and their young person in a developmental context with the hope of a positive outcome. Each of these themes are congruent with themes that have consistently been reported by BEST-Plus participants, from the initial implementation of the program over a decade ago [18]. 

In terms of evaluating the BEST-Plus groups within the RCT, these findings suggest that parents received many of the key features of the intervention as presented in the BEST-Plus manual and training materials. The consumer feedback consistently suggests that what parents received corresponds closely with what the manual intended. In this sense, the findings presented add to the probability that the intervention was delivered in a way that was consistent with the treatment manual. These findings also add to the evidence that the BEST-Plus training and supervision provide an effective transmission of the program logic to a diverse range of mental health clinicians. The effective implementation of the program logic can be seen in terms of the changes in parenting style reported by a majority of the focus group participants. However, given the small sample who participated in these focus groups, it remains unknown the degree to which this finding can be generalised to the full RCT sample, or to other groups who undergo BEST-Plus programs. Often parents had expected a change in the behaviour of their young person through participation in the group but generally they found the greatest change was in how they viewed situations and how they responded to their young people. This illustrates the systemic mechanism through which change is often achieved in family-based interventions.

The DFO study was designed as a multicentre trial, and included a wide range of referrals from clinical services, community services, and community organisations such as schools; to further enhance generalizability of findings. The study design was initially developed under the expectation that the referred youth would be motivated to attend a treatment, and that their parents would enter the treatments if they were randomly allocated to the family intervention. Unexpectedly, many of the referrals to the DFO study came from concerned parents where the young person was unwilling to initially engage in a treatment program for their depression. Rather than excluding these families, the research team decided to allow the parents to access the only possible treatment (BEST-Plus, as it can be completed with the parents alone or with whole families), and to evaluate the outcomes for these families following the program. This was an attempt to prevent the exclusion of a relevant and large cohort of needy families, who appear to be underresearched and underserviced under the current Australian mental health system, given the reluctance of the young person to attend a standard treatment [22, 25].

The current study has a number of implications for the effective implementation of family-based interventions for youth mental health. Diagnostically, the current sample shows considerable heterogeneity in youth mental health issues, with both internalizing and externalizing profiles represented. This reflects the common referral patterns of clinical practice. Typically, referral to mental health services in this age group is initiated by parents, or at the very least strongly encouraged by parental support. Both intake and initial assessment procedures in youth mental health could thus benefit from a stronger family focus to reflect this common circumstance. The other key finding from our study is the clear capacity to achieve considerable transformation of family functioning within a relatively brief, intensive, and highly structured group format. Feedback from parents suggests that developmental themes of separation individuation remain highly salient, and that many families are receptive to interventions designed to facilitate the transition from adolescence to early adulthood.

One of the key outcomes of the DFO study was that the BEST-Plus model was modified to encourage the identified youth to attend the program with their parents and siblings, and to provide support for families whose young person presents more of an internalizing profile (where depression and anxiety are the dominant presenting issues). Previous versions of BEST tended to focus more on externalizing problems associated with substance use and employed behavioural management techniques and boundary setting. Focus group participants as well as the research team were also concerned by the high rate of nonparticipation of the young people in the second half of the BEST-Plus group. However, nonattendance was mitigated by parents engaging their children in discussion about the group and the flow on effect by their changed parenting styles and view of their situation. Many participants would also have liked some continuation of the group because they found the parental support valuable. They also felt that this sort of program should become more preventative, acting as an early intervention in schools. The Deakin Family Options program, and in particular, the BEST-Plus group, led to a number of positive changes according to the focus group participants.

 Based on the findings in the DFO study, and the feedback from consumers described in the current paper, the BEST-Plus program has subsequently been extended to a third stage of development, known as BEST-MOOD [26]. The BEST-MOOD program has integrated much of the feedback presented in this paper and is aimed at addressing the problem of engaging young people in mental health treatments via the family system, and delivering relevant and effective interventions in the community. Notably there was a departure from the stated intention of the BEST-Plus manual in terms of young people directly impacted by mental health issues attending the youth component of the groups in the DFO study. This was consistently adopted across the interventions in the DFO trial and then integrated into the current revised BEST-MOOD model, so it is less of a “limitation” as perhaps a development that occurred within the DFO trial.

There are a number of important limitations to the present study that should be considered when appraising its findings. The findings do not explore the perspective of families that did not engage and thus may miss an important contrary perspective. There was limited information available from young people whose parents were in BEST-Plus and the findings are based mostly on the views of parents. It is also notable that the BEST-Plus facilitator was in many cases also focus group leader which may have biased discussion in a positive direction—and yet it is notable that significant suggestions and criticisms of the program were still forthcoming. In general, it should be noted that while a focus group is an effective way of gathering a large number of views, there is always the possibility that a large group may not allow dissenting voices to be expressed.

## 6. Conclusions

There is a clear recognition by governments that the cost of depression is high and effective depression prevention and early intervention programs are likely to be worth implementing (1). However, to implement these plans of action, there needs to be substantial investment to build the knowledge and infrastructure for prevention and early intervention including research capacity, prevention, and early intervention program development, evaluation and implementation frameworks. Our experience with gaining qualitative evaluation of consumer experiences within a RCT convinces us that such evaluation ought to be routinely used for gathering and analysing participant feedback in order to improve treatments. 

## Figures and Tables

**Table 1:** Demographic features of participants in the consumer reference groups (*n* = 21).

Demographic feature	*M*	SD
Age of participant (yrs)	48.8	9.04

	*n*	%

Family member		
Mother	12	4.8
Father	8	57.1
Youth (male)	1	38.1
Marital status		
Married	13	61.9
Divorced	3	14.3
Separated	1	4.8
Family annual income		
Less than $50000	3	14.3
$50000–$80000	3	14.3
Over $80000	9	42.9
Missing	6	28.6
Level of education		
Completed year 10	2	9.5
TAFE diploma or certificate	5	23.8
Undergraduate degree	3	14.3
Postgraduate degree	1	4.8
Other	3	14.3
Missing	7	33.3
Number of BEST-Plus sessions attended		
1	1	4.8
3	2	9.5
4	1	4.8
5	1	4.8
6	1	4.8
7	10	47.6
8	5	23.8

**Table 2:** Cross tabulation of engagement in treatment and type of family member.

	Did not engage in treatment	Engaged in treatment
Identified Youth	47 (66%)	24 (34%)
Sibling	9 (69%)	4 (31%)
Father	6 (21%)	23 (79%)
Mother	22 (31%)	48 (69%)

**Table 3:** Results of consumer satisfaction survey for parents participating in BEST-Plus (*n* = 20).

Satisfaction							Range	
					*M*	SD	High	Low
With the intervention received					7.66	1.58	10	3
With any improvements in your family/home life					6.64	1.65	10	3
Overall satisfaction with program					8.30	1.49	10	4

							*n*	%

Felt the program adequately addressed your needs						(yes)	12	57.1
						(somewhat)	8	38.1
Would recommend participating in this program to a friend						(yes)	20	95.2
Is still implementing the skills and knowledge from the program						(yes)	20	95.2
